# Sleep duration links to biological aging clocks: when sleep gets tough, aging gets going

**DOI:** 10.1038/s41392-026-02997-7

**Published:** 2026-09-07

**Authors:** Despina Tawadros, Melek Canan Arkan

**Affiliations:** 1 Institute for Tumor Biology and Experimental Therapy, Georg-Speyer-Haus, Frankfurt/Main, Germany; 2 Frankfurt Cancer Institute, Goethe University Frankfurt, Frankfurt/Main, Germany; 3 German Cancer Consortium (DKTK) and German Cancer Research Center (DKFZ), Heidelberg, Germany

**Keywords:** Predictive markers, Biochemistry

In a recent large-scale study of biological ageing clocks published in *Nature*, the MULTI Consortium now shows sleep, often treated as a lifestyle habit, is a measurable sign of temporal biology. Their work suggests that deviations from optimal sleep duration are associated with accelerated biological aging across multiple organs and that healthy sleep may be one of the most practical and scalable ways for promoting longevity and reducing chronic disease risk.^1^

The study is distinguished by its scale and multidimensional assessment of aging biology. Rather than relying on a single aging biomarker, the authors examined 23 biological aging clocks derived from MRI, plasma proteomics, and metabolomics in 500,000 individuals aged 37–84 years. They identified a U-shaped relationship between sleep duration and biological age gaps, with the lowest age gaps observed at approximately 6.4–7.8 h of sleep, depending on organ and sex. This pattern was significant in 9 of 23 clocks and was not confined to the brain. Indeed, associations were also observed in heart, pulmonary, hepatic, immune, skin, endocrine, adipose, and pancreatic aging clocks, suggesting that sleep duration captures broad cross-organ biological aging rather than isolated neurobiological decline.

This organ-level perspective is timely. Recent work from the same group has shown that aging can be mapped across several organs, and that organ age gaps relate to proteins, metabolites, genetic architecture, disease risk, mortality, and clinical trial trajectories.^2^ This emphasizes that organs age at different rates and that premature aging in one organ may influence aging in connected organs. Thus, the sleep study is not only about sleep duration as it places sleep within a complex systems biology of aging, where disrupted temporal biology may leave detectable signatures across multiple tissues.

**Fig. 1:**
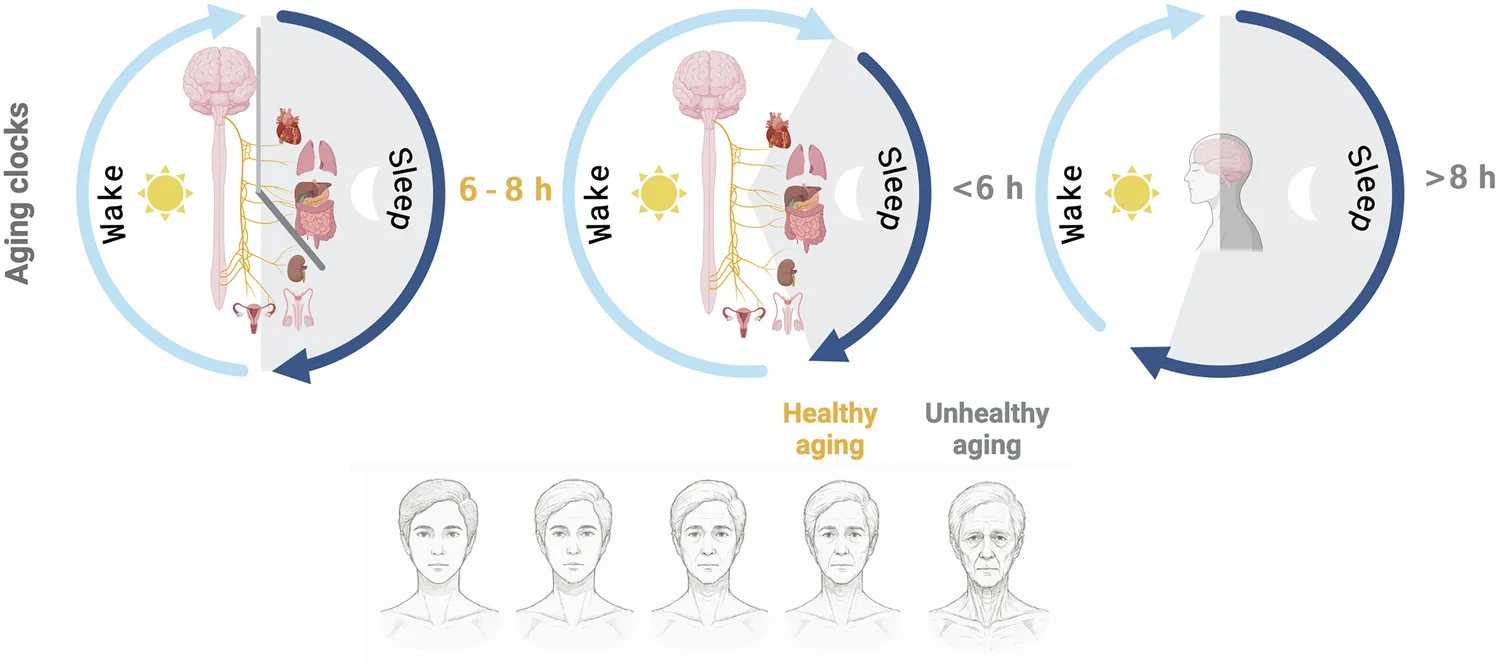
Disrupted sleep duration patterns compared to normal sleep duration impact human aging and disease across the lifespan. Both short (< 6 h) and long (> 8 h) sleep is linked to elevated biological aging burden across many organ systems. This close relationship between altered sleep duration and multi-organ ageing clocks leads to increased systemic disease outcomes and mortality risk. Created with BioRender

The clinical implications are equally important. Short sleep (< 6 h) and long sleep (> 8 h) were both linked to higher disease risk and all-cause mortality. The authors identified 153 significant associations between abnormal sleep duration and future disease endpoints, with short sleep showing particularly strong links to mental health, metabolic disease, cardiovascular disease, pulmonary disease, and digestive disorders. Yet short and long sleep operated through different pathways. Short sleep showed a more direct association with late-life depression, potentially through acute stress, immune dysregulation, sympathetic activation, and hypothalamic-pituitary-adrenal axis signaling. Long sleep, by contrast, appeared more indirectly linked to neuropsychiatric outcomes, with brain and adipose aging clocks partly contributing to the pathway to late-life depression (Fig. 1).

The biology of sleep offers a possible mechanistic bridge. A recent review of the oscillatory biology of sleep argues that sleep reorganizes major neuromodulators including norepinephrine, acetylcholine, serotonin, dopamine, and histamine into coordinated brainwide rhythms.^3^ These oscillations help structure sleep microarchitecture, vascular dynamics, cerebrospinal fluid flow, and glymphatic clearance of metabolic waste. Disruption of these rhythms through aging, stress, cardiovascular disease, psychiatric disease, medications, or substance use may impair clearance of neurotoxic proteins such as amyloid-β and tau. Thus, sleep is not simply behavioral rest but rather an active physiological state that synchronizes neural, vascular, immune, metabolic, and clearance programs.

This mechanistic insight may also explain why sleep disruption associates with biological aging clocks. Recent transcriptomic studies of mammalian aging and mortality identified conserved molecular modules involving inflammation, interferon signaling, mitochondrial function, chromatin modification, extracellular matrix organization, metabolism, and stress responses.^4^ As a matter of fact, many of these pathways are sensitive to sleep and circadian disruption. Sleep may therefore influence aging not through a single route, but by altering rhythmic coordination of inflammatory, metabolic, vascular, neuroendocrine, and repair programs.

The study has several limitations. In a cohort of 500,000 individuals, small differences in the biological age gaps can reach statistical significance. It reports p-values and curve complexity, but not variance explained or effect sizes, making it unclear how much variation in organ aging is accounted for by sleep duration. The U-shaped curves were also selected from candidate smoothing parameters and distributional families using the same data later used to test significance, raising the possibility of in-sample model selection inflating significance. Moreover, the genetic instruments were binary, short or long sleep versus normal, whereas the central finding is a continuous U-shaped relationship.

Other limitations concern sleep and biomarker measurements. Sleep duration was self-reported rather than objectively measured by actigraphy or polysomnography. The study did not capture sleep quality, fragmentation, timing, sleep architecture, or sleep regularity. Proteomic and metabolomic profiles also fluctuate around the day, and single static sampling may imperfectly represent stable biological aging, especially without fully accounting for illness, medication, diet, hydration, or transient exposures. The study also did not directly assess circadian misalignment, shift work, light exposure, chronotype, or meal timing. Future studies should combine longitudinal objective sleep phenotyping with repeated molecular and imaging clocks to distinguish transient biological noise from durable aging trajectories.

This is especially important because sleep changes with age. Older adults often sleep less deeply and less continuously, not necessarily because they need less sleep. Aging weakens the master clock in the suprachiasmatic nucleus, shifts sleep-wake rhythms earlier, reduces restorative slow-wave sleep, and dampens melatonin signaling. Comorbidities, medications, nocturia, pain, sleep apnea, depression, and reduced daytime light exposure further fragment sleep. Thus, short sleep in older adults may reflect both modifiable sleep disruption and underlying disease, whereas long sleep may signal fatigue, inflammation, frailty, or subclinical illness rather than restorative rest.

But what about the younger population? Although this study focused on middle-aged and older individuals, its implications extend earlier in life. Younger generations are increasingly exposed to irregular sleep schedules, artificial light at night, late-night eating, shift work, and social jet lag, which could possibly promote earlier tissue aging. This raises the hypothesis that disrupted sleep may not only reflect poor health, but also embed modern lifestyle exposures that contribute to earlier disease vulnerability. Testing this will require longitudinal cohorts integrating sleep phenotyping, biological aging clocks, circadian disruption, inflammatory and metabolic biomarkers, immune surveillance, and disease outcomes, especially as early-onset malignancies continue to rise.

Despite limitations, this study provides strong cues for translational aging research. Its key contribution is not the claim that a precise number of sleep hours will causally slow aging in every person, but that sleep duration tracks with multi-organ and multi-omic biological aging and may serve as a biomarker. It will be critical to incorporate sleep measures into risk stratification, aging-clock studies, and intervention trials using wearables,^5^ actigraphy, sleep diaries, light-exposure profiles, and meal timing. The goal is not simply to fix sleep duration, but to restore rhythmic coherence across behavioral, endocrine, immune, metabolic, and molecular systems.

Until precision sleep becomes routine in longevity research, the message remains simple: protect your biological time. Maintain your regular daily rhythms, unplug before bed, keep the room dark, and get a good night’s zzzleep.
